# Transcutaneous electrical acupoint stimulation for intercostobrachial nerve syndrome after breast cancer surgery: a randomized controlled trial protocol

**DOI:** 10.3389/fgwh.2026.1826269

**Published:** 2026-06-19

**Authors:** Qilian Xia, Linna Wu, Yuhuan Sun, Shumo Li, Xingtai Hu, Zekai Liang, Ziyou Zhang, Aimei Jiang, Rong Zhao

**Affiliations:** 1 Yunnan Provincial Hospital of Traditional Chinese Medicine, Kunming, Yunnan, China; 2 The First Clinical Medical School, Yunnan University of Chinese Medicine, Kunming, Yunnan, China; 3 The First Affiliated Hospital, Kunming Medical University, Kunming, China; 4 Department of Basic Medical Sciences, Shanghai Medical College, Fudan University, Shanghai, China; 5 The Second Clinical Medical School, Yunnan University of Chinese Medicine, Kunming, Yunnan, China

**Keywords:** breast cancer, intercostobrachial nerve syndrome, randomized controlled trial, studyprotocol, surgery, transcutaneous electrical acupoint stimulation (TEAS)

## Abstract

**Introduction:**

Intercostobrachial nerve (ICBN) syndrome is a common peripheral sensory nerve injury following axillary surgery for breast cancer. It is characterized by persistent numbness and burning pain extending from the affected axilla to the medial aspect of the upper arm, as well as restricted shoulder joint mobility, which severely impacts postoperative recovery and quality of life. Current interventions primarily focus on symptomatic analgesia and functional exercises, lacking standardized external treatment protocols to promote nerve repair. Transcutaneous electrical acupoint stimulation (TEAS) combines the advantages of acupuncture and transcutaneous electrical stimulation. It is non-invasive and has high patient acceptance. Preliminary studies suggest it can promote axonal regeneration and myelin formation, yet scientific evidence-based evidence for ICBN syndrome is lacking. This study aims to systematically evaluate the therapeutic effects and safety of TEAS on ICBN syndrome through a prospective, randomized, sham-controlled trial, providing evidence-based medical evidence for clinical practice.

**Methods and analysis:**

This prospective, randomized, sham-controlled clinical trial will be conducted at the Yunnan Provincial Hospital of Traditional Chinese Medicine. We plan to recruit 200 patients with ICBN syndrome after breast cancer surgery. Participants will be randomly assigned in a 1:1 ratio to either the TEAS group or the Sham TEAS group, with 100 cases in each group. Both groups will start receiving treatment on the first postoperative day, administered once every other day. Ten sessions will constitute one course, for a total of three courses. The primary outcome measure is CMS. Secondary outcome measures include MMT, EMG, VAS, SWMT, FACT-B, SF-36, and TCM-SD. Adverse events will be recorded throughout the study.

**Clinical Trial Registration:**

https://itmctr.ccebtcm.org.cn/mgt/project/view/2041939715654978881, identifier, ITMCTR2024000864.

## Highlights

- This study employs a relatively large sample size (*N* = 200) and utilizes stratified randomization based on the surgical approach (ICBN-sparing status). This design significantly enhances statistical power and ensures baseline balance and comparability between groups.
- A 12-month follow-up period has been established. This design fully accounts for the physiological characteristics of slow peripheral nerve regeneration and functional recovery, enabling the effective capture of the intervention's long-term efficacy.
- The outcome measures integrate objective neurophysiological metrics (e.g., EMG, SWMT) with subjective symptom scores (e.g., CMS, VAS), providing objective and direct evidence for nerve repair.
- As a single-center study, the generalizability of the findings may be influenced by specific medical settings and geographic factors.
- While strict exclusion criteria (e.g., excluding patients with a history of chronic pain or long-term analgesic use) enhance internal validity, they may limit the applicability of the results to a broader patient population with complex comorbidities.

## Introduction

Breast cancer is the most common malignant tumor among women worldwide. Currently, the most widely applied treatment model is a comprehensive treatment modality centered on surgery, covering adjuvant therapies such as radiotherapy and chemotherapy (1). The intercostobrachial nerve (ICBN) is the lateral cutaneous branch of the second intercostal nerve. It pierces the posterior border of the serratus anterior muscle from the chest wall, enters the axilla, joins the medial brachial cutaneous nerve, and innervates the skin of the medial upper arm and the subscapular region. During sentinel lymph node biopsy or axillary lymph node dissection, the ICBN is susceptible to traction, clamping, or transection. This leads to pain, numbness, burning sensations, or hypoalgesia and thermohypesthesia in its innervated areas (the affected axilla, medial upper arm, and subscapular region), as well as varying degrees of pectoral muscle atrophy, upper limb movement disorders, and functional decline on the affected side. These symptoms are clinically collectively referred to as “ICBN Syndrome” (2). It has been reported that the incidence of postoperative sensory abnormalities in patients without ICBN preservation is 36.4%. Even with modified dissection preserving the nerve, 2%–4% of patients still experience persistent sensory disturbances, and symptoms in some patients do not recover even after 12 months (3). In recent years, surgical strategies preserving the ICBN have gained widespread recognition and support (4). However, regardless of the surgical method used, some patients still present with ICBN syndrome postoperatively. This not only severely affects the patients' quality of life but is also a significant factor hindering their rehabilitation exercises and inducing anxiety and depression (5).

Currently, clinical interventions for ICBN syndrome primarily consist of analgesic medications and functional exercises, lacking standardized external treatment protocols aimed at nerve repair (6). Although Beshoy Nabil Fam et al. (7) reported that pulsed radiofrequency combined with steroid injection can alleviate related symptoms, the sample size was small, the procedure is invasive, and it is difficult to generalize. In the field of Traditional Chinese Medicine (TCM), although there are case reports of “Songjin Liluo” (tendon-relaxing and collateral-regulating) manipulation combined with external application of Shuangbai Powder improving postoperative numbness (8), verification through randomized controlled trials is lacking, and the level of evidence is limited.

TCM possesses unique advantages in nerve injury repair and the treatment of sensory disturbances such as pain and numbness (9). Studies have shown that electroacupuncture can induce Schwann cell proliferation, promote myelination and nerve fiber regeneration, and improve nerve ultrastructure, thereby accelerating peripheral nerve functional recovery (10). Transcutaneous electrical nerve stimulation (TENS) activates residual axons through microcurrents, significantly increases the diameter of regenerating axons, and accelerates axonal sprouting and myelination (11). Simultaneously, it improves local microcirculation, rebuilds “nerve-muscle” excitation-contraction coupling, induces rhythmic muscle contraction, and achieves dual structural and functional repair (12). TEAS integrates the advantages of TENS and acupuncture. Characterized by targeted acupoint selection, simple operation, non-invasiveness, safety, and high patient compliance, it has been proven effective in intervening in perioperative pain, numbness, and functional dysfunction (13). However, although numerous randomized clinical trials have described the effectiveness of TEAS in treating various diseases, there is still a lack of high-quality evidence-based evidence for its application in ICBN syndrome following sentinel lymph node biopsy or axillary lymph node dissection for breast cancer. This study aims to systematically observe the therapeutic effect of TEAS on ICBN syndrome through a randomized controlled clinical trial, intending to provide a scientific basis for the promotion of external TCM treatments in the rehabilitation of postoperative nerve injury in breast cancer.

## Methods and analysis

### Study design

**Figure 1:**
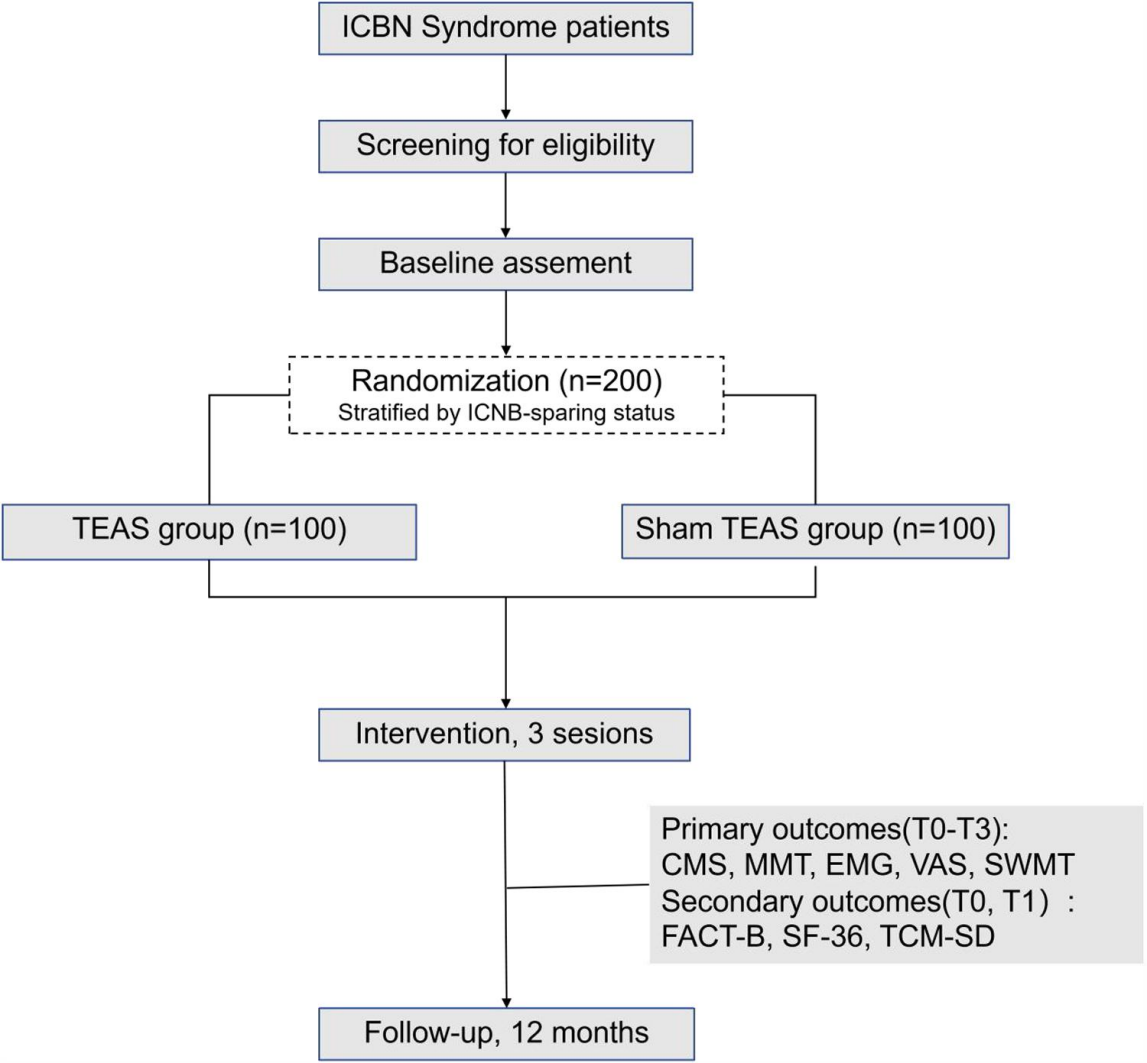
Trial flow diagram. ICBN, intercostobrachial nerve; TEAS, transcutaneous electrical acupoint stimulation; CMS, Constant–Murley score; MMT, manual muscle test; EMG, electromyography; VAS, visual analogue scale; SWMT, Semmes-Weinstein monofilament test; FACT-B, functional assessment of cancer therapy-breast; SF-36, 36-Item short form health survey; TCM-SD, traditional Chinese medicine syndrome score.

**Figure 2:**
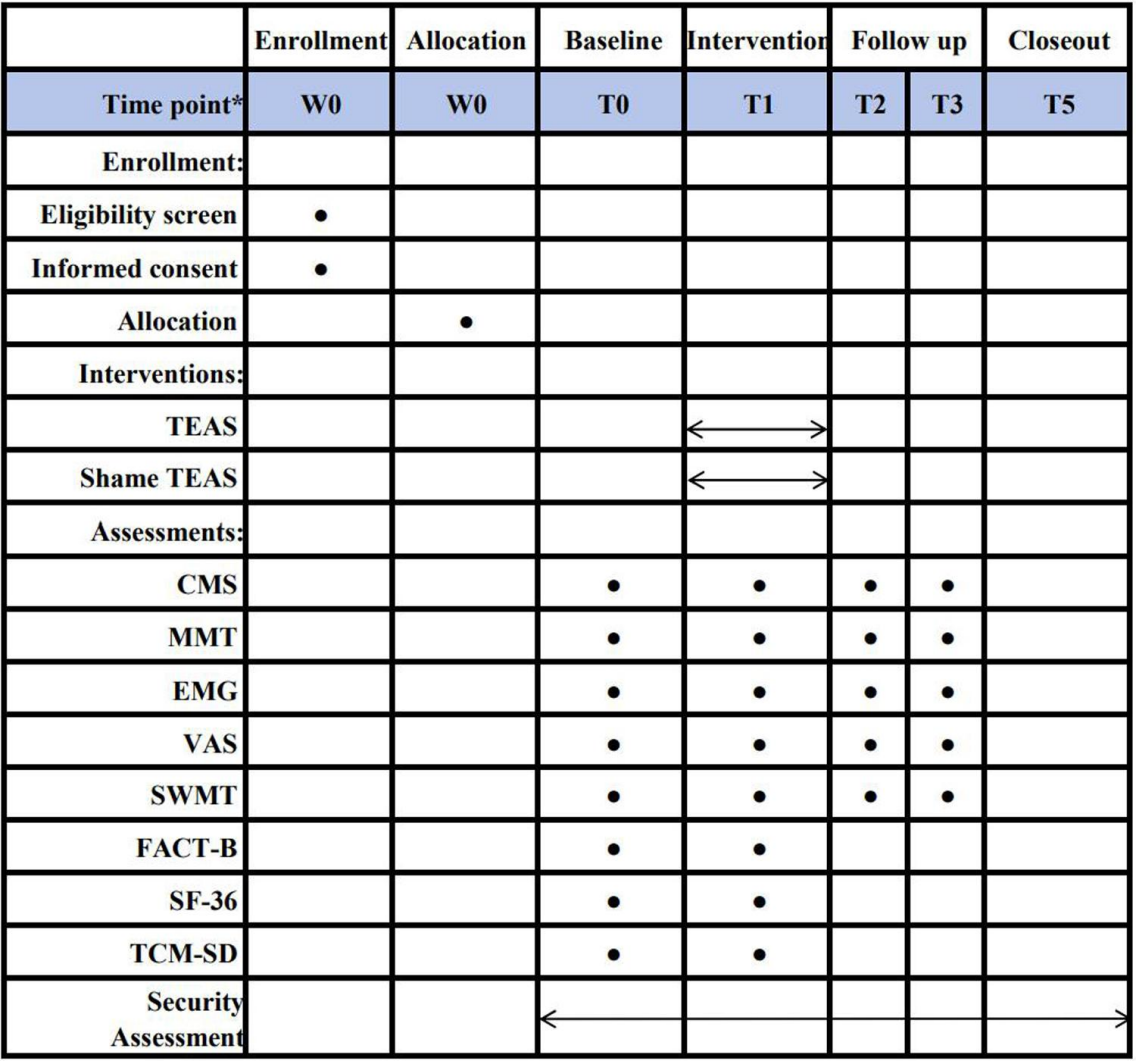
Schedule of enrolment, interventions and assessments.

This study is a prospective, single-center, randomized, sham-controlled, parallel-group clinical trial designed to evaluate the efficacy of TEAS for ICBN Syndrome following breast cancer surgery. We plan to recruit 200 participants. All enrolled individuals will be stratified based on the type of surgery (i.e., ICBN-sparing status) and randomly assigned in a 1:1 ratio to either the TEAS group or the sham TEAS group. The study adheres to the reporting guidelines of the Consolidated Standards of Reporting Trials (CONSORT) and the Standards for Reporting Interventions in Clinical Trials of Acupuncture (STRICTA) and has been registered at ccebtcm.org.cn (Registration No.: ITMCTR2024000864). The trial flow is illustrated in Figure 1, and the schedule for recruitment, intervention, and assessment is presented in Figure 2.

### Recruitment

From January 2025 to January 2027, patients diagnosed with ICBN syndrome after breast cancer surgery who meet the inclusion criteria will be consecutively recruited and registered at the Yunnan Provincial Hospital of Traditional Chinese Medicine. The recruitment period is 24 months. Before the study begins, staff members will fully explain the trial details, potential risks, and benefits to the patients. Eligibility screening and randomization will be conducted after written informed consent is obtained.

### Participants

#### Diagnostic criteria

Breast cancer diagnosis: Performed in accordance with the Guidelines and Norms for the Diagnosis and Treatment of Breast Cancer (2024 Edition) issued by the China Anti-Cancer Association (14).

Diagnosis of post-breast cancer surgery ICBN syndrome: Clinical manifestations include postoperative stiffness of the shoulder joint on the affected side, muscle adhesion, muscle atrophy, limited range of motion of the shoulder joint, paresthesia or sensory loss in partial areas, muscle weakness, rapid fatigue after exercise, and fine motor dysfunction.

#### Inclusion criteria

(1) Meet the diagnostic criteria for breast cancer (15) and postoperative ICBN syndrome;
(2) Breast cancer patients who underwent unilateral total mastectomy or breast-conserving surgery combined with axillary lymph node dissection;
(3) Patients with unilateral non-metastatic breast cancer;
(4) Aged between 25 and 75 years;
(5) Receiving follow-up treatment at our hospital after surgery;
(6) Conscious, cooperative with treatment, and able to comply with follow-up during the study period;
(7) Estimated survival time > 12 months.

#### Exclusion criteria

(1) Patients whose final histological examination does not confirm breast cancer;
(2) Patients who received other treatments preoperatively, or have a history of chronic pain and long-term use of analgesics or neurotrophic drugs;
(3) History of previous breast surgery;
(4) Preoperative arm movement disorders or neuropathy;
(5) Patients receiving other adjuvant therapies.

#### Sample size

This study employs a superiority test for two independent sample means. The sample size calculation is based on the change in the Constant-Murley Score (CMS), the primary outcome measure, from baseline (T0) to the end of treatment (T1). Based on relevant prior literature and clinical observations, the recovery of shoulder joint function in patients with post-breast cancer ICBN syndrome often experiences a significant decline or stagnation in the early postoperative period (T1 phase) due to surgical trauma and subsequent adjuvant therapies (such as radiotherapy and chemotherapy). The recovery process is not a smooth linear ascent; rather, it is significantly influenced by treatment timing and exhibits marked fluctuations (15). Furthermore, considering the potential non-specific effects in the sham TEAS group, we established robust estimation parameters: the expected mean improvement in CMS score after intervention is 18.5 ± 15.0 for the trial group and 11.0 ± 15.0 for the control group (i.e., expected between-group difference δ = 7.5, pooled standard deviation σ = 15.0). With a two-sided significance level of α = 0.05 and a power of 1‒β = 0.90, the calculated required sample size is 85 cases per group using statistical software. Considering the long follow-up period (12 months) and the potential for participant dropout due to subsequent radiotherapy or chemotherapy, we adjusted for a 15% attrition rate. Consequently, the final total sample size was determined to be 200 cases, with 100 cases in each group.

#### Randomisation and blinding

Eligible patients will be randomly assigned in a 1:1 ratio to receive either TEAS or sham TEAS. Independent staff will assist in establishing and managing the randomization database. Both the observation and treatment groups will receive treatment in enclosed units during each session. Participants, outcome assessors, statisticians, and data analysts will remain blinded to the allocation. Although the acupuncturists will be aware of the allocation, they will not participate in the outcome assessment or data analysis throughout the study.

#### Intervention

All patients who develop neuropathic pain and sensory abnormalities will receive standardized basic symptomatic treatment. The specific regimen is: gabapentin (starting dose 300 mg/day, adjustable within 900–1,800 mg/day under medical guidance based on tolerability and pain control, maintained during the study period), mecobalamin (0.5 mg three times daily), and oryzanol (10 mg three times daily). No other types of analgesics (e.g., NSAIDs, opioids) or neurotrophic drugs are permitted during the study period. If a patient requires rescue medication due to severe pain, the drug name, dose, timing, and reason will be recorded and adjusted for as a covariate in the final analysis. Both groups will receive routine postoperative functional exercises and standard nursing care for breast cancer.

All treatments will be performed in a clean, quiet treatment room. Patients will be informed that “there may be no obvious sensation during the treatment”. Operators will be licensed practitioners with ≥10 years of clinical experience who have undergone standardized training in TEAS operation.

#### TEAS group (treatment group)

Device: KD-2A Transcutaneous Electrical Nerve Stimulation Therapeutic Apparatus (Manufacturer: Beijing Yaoyang Kangda Medical Instrument Co., Ltd.).

Acupoint Selection: Jianzhen (SI9), Tianzong (SI11), T1–T3 Jiaji (Ex-B2), Zusanli (ST36), and Sanyinjiao (SP6).

Acupoint Location: Referencing the Standard International Acupuncture Nomenclature (90/8579-Atar-8000) proposed by the WHO, the specific locations are as follows:

① Jianzhen (SI9): Located posterior and inferior to the shoulder joint, 1 cun directly superior to the posterior end of the axillary fold. ② Tianzong (SI11): In the scapular region, in the depression of the center of the infraspinous fossa, level with the 4th thoracic vertebra. ③ Jiaji (Ex-B2): On the back and lower back, 0.5 cun lateral to the posterior midline, below the spinous processes of the 1st thoracic vertebra to the 5th lumbar vertebra (17 points per side). In this study, the 3 points on the affected side below the spinous processes of T1–T3 are selected. ④ Zusanli (ST36): On the anterior aspect of the lower leg, 3 cun below Dubi, one finger-breadth (middle finger) from the anterior crest of the tibia. ⑤ Sanyinjiao (SP6): On the medial aspect of the lower leg, 3 cun above the tip of the medial malleolus, posterior to the medial border of the tibia.

Position: Patients will take a seated position with the shoulder, back, and lower limbs exposed.

Operation: For Tianzong, Jianzhen, and T1–T3 Jiaji on the affected side: Frequency 50 Hz, pulse width 50 μs, output pulse 5 Hz, for 30 min. For bilateral Zusanli and Sanyinjiao: Frequency 50 Hz, pulse width 50 μs, output pulse increasing from 2 to 100 Hz, for 30 min.

Course of Treatment: Treatment will be initiated on the first postoperative day and administered once every other day. Ten sessions constitute one course, with a 3–5 day break between courses. A total of three consecutive courses (30 sessions in total) will be administered.

#### Control group (sham TEAS)

The electrode placement will be identical to that of the TEAS group. To mimic the sensation of real treatment, the intensity will be adjusted to the sensory threshold and then immediately reduced to 0 mA within 15 s, ensuring no effective pulse output throughout the session. Our team has verified the feasibility of this sham stimulation method in previous clinical practice. The device's indicator lights and buzzers will remain active. Furthermore, we will evaluate the success of blinding among participants using a blinding assessment questionnaire (e.g., Bang's Blinding Index) at the end of treatment (at time point T1) to validate the effectiveness of our sham control.

### Outcome measurement

#### Measurement time points

T0 (Pre-treatment): Before intervention on the 1st postoperative day.

T1 (Post-treatment): After 3 courses of treatment.

T2 (Follow-up): 6 months after surgery.

T3 (Follow-up): 12 months after surgery.

#### Primary outcomes

CMS (Constant-Murley Score): Used to assess the overall functional status of the shoulder joint. It includes Pain (P, 15 points), Range of Motion (R, 40 points), Activities of Daily Living (A, 20 points), Muscle Strength (M, 25 points), and local form of the shoulder joint (F). The total score ranges from 0 to 100, with higher scores indicating better function. By combining subjective and objective measures, this index comprehensively evaluates the actual benefits in daily life for participants after TEAS intervention. This assessment will be conducted at T0, T1, T2, and T3.

#### Secondary outcomes

MMT (Manual Muscle Testing): The Medical Research Council (MRC) 0–5 grading scale is used to assess the contraction level of key muscle groups in the affected upper limb (e.g., deltoid, pectoralis major). A higher grade indicates greater muscle strength. This indicator aims to evaluate the overall recovery of muscle strength in the shoulder girdle region after the relief of “pain-movement inhibition” caused by ICBN injury. This assessment will be conducted at T0, T1, T2, and T3.

EMG (Electromyography): This focuses on detecting the Sensory Conduction Velocity (SCV) and Sensory Nerve Action Potential (SNAP) amplitude of the axillary-arm segment of the ICBN. Since anatomical studies confirm that the ICBN is a pure sensory nerve and does not innervate any muscle movement, this study will not measure motor nerve conduction parameters. The recovery of SNAP amplitude directly reflects the sprouting of residual nerve axons and the integrity of the myelin sheath, serving as key objective “hard evidence” to determine whether TEAS can promote functional nerve recovery. This assessment will be conducted at T0, T1, T2, and T3.

VAS (Visual Analogue Scale): A 10 cm linear segment is used, with one end representing “no pain” and the other representing “the most severe pain imaginable”. Patients mark the line according to their current pain intensity. As a supplement to the CMS pain module, VAS provides more sensitive continuous quantitative data, allowing for a more precise capture of the analgesic effects of TEAS at different follow-up points. This assessment will be conducted at T0, T1, T2, and T3.

SWMT (Semmes-Weinstein Monofilament Test): Monofilaments of varying thickness are used to determine the tactile threshold of the skin on the medial side of the affected upper arm. Smaller gram force of the monofilament indicates higher tactile sensitivity. This indicator is specifically used to quantify the degree of sensory loss in the area innervated by the ICBN, forming a complete corroborative chain of microstructure and macro-sensation together with the electrophysiological data from EMG. This assessment will be conducted at T0, T1, T2, and T3.

FACT-B (Functional Assessment of Cancer Therapy-Breast): Assessed at T0 and T1 to evaluate quality of life. It contains a general module and a breast-specific module, with a total score of 0–144; higher scores indicate better quality of life.

SF-36: Assessed at T0 and T1 to evaluate overall health. It is divided into 8 domains with 36 items, with a total score of 0–100; higher scores indicate better health status.

TCM-SD (TCM Syndrome Score): Assessed at T0 and T1. Symptoms such as limb pain, constipation, thirst, and fatigue are scored as 0–3 based on None/Mild/Moderate/Severe.

#### Safety assessment

Patient safety will be continuously monitored during the study and summarized at the end of the research. Safety endpoints include laboratory indicators (blood routine examination, liver function, renal function, and electrocardiogram), adverse events (AEs), and serious adverse events (SAEs).

### Data collection and management

All source data will be uniformly recorded in the Case Report Forms (CRF) and entered into the study's data management system by the researchers. All personally identifiable information (such as names, ID numbers, phone numbers, etc.) will be anonymized. For participants who discontinue treatment or deviate from the protocol, researchers will make every effort to collect outcome data during the follow-up period. The database will be locked after the last participant completes the final follow-up and double verification is performed; once locked, no personnel will be able to modify the data. All participant files will be encrypted, centrally managed by the research center, and kept strictly confidential to ensure data security and traceability.

### Quality control

The trial design has been evaluated and refined by experts in acupuncture, breast surgery, statistics, and methodology. Any modifications to the trial will be reported to the Ethics Committee of Yunnan Provincial Hospital of Traditional Chinese Medicine. Any changes to the database will be recorded in the CRF.

### Data analysis

All statistical analyses will be performed using SPSS 26.0. All statistical tests will be two-sided, with *P* < 0.05 considered statistically significant.

Definition of Analysis Sets: The primary efficacy analysis will be based on the Intention-to-Treat (ITT) set, which includes all randomized participants who have received at least one treatment. The safety analysis will be based on the Safety Set (SS).

Baseline Description: Continuous variables conforming to a normal distribution will be expressed as Mean ± Standard Deviation (SD), while non-normally distributed variables will be expressed as Median (Interquartile Range, IQR). Categorical variables will be expressed as frequencies and percentages (%). Baseline balance between groups will be compared using the independent samples *t*-test, Mann–Whitney *U*-test, or Chi-square (*χ*^2^) test.

Primary Outcome Analysis (Linear Mixed-Effects Model): A Linear Mixed-Effects Model (LMM) will be constructed for the repeated measurements of the sole primary outcome measure, the Constant-Murley Score (CMS), at four time points (T0, T1, T2, T3). The model will use the observed CMS values at each time point as the dependent variable, with group (trial group vs. control group), time (categorical variable), and the “group × time” interaction term as fixed effects, and subject ID as a random effect. Baseline CMS score, surgical approach (e.g., whether the ICBN was preserved), and study center will be included as covariates. If the “group × time” interaction effect is significant, *post-hoc* comparisons between groups at each time point will be further performed using the LSD method or Bonferroni correction.

Secondary Outcome Analysis: For secondary outcome measures (MMT, EMG, VAS, SWMT, FACT-B, SF-36, TCM-SD), appropriate statistical methods will be applied based on data distribution. For continuous variables, independent samples *t*-test or repeated-measures ANOVA will be used if normality is satisfied; otherwise, the Mann–Whitney *U*-test or generalized estimating equations will be used. Categorical variables will be analyzed using the *χ*^2^ test or Fisher's exact test.

Handling of Missing Data: For the primary outcome CMS, the Linear Mixed-Effects Model assumes data are Missing At Random (MAR) and utilizes all available data for parameter estimation. To assess the robustness of the results, sensitivity analyses will be conducted, including: (1) multiple imputation (5–10 imputations) followed by re-analysis; (2) best-case and worst-case scenario analyses for key variables with a missing rate exceeding 20%. Reasons for participant dropout will be documented in detail and reported in the paper.

Safety Analysis: The incidence of adverse events will be calculated, and comparisons between groups will be performed using Fisher's exact test.

### Trial status

The trial is currently in the recruitment phase. As of August 2025, 10 participants have been enrolled.To ensure the target sample size of 200 participants, the recruitment period was extended from 12 to 24 months (January 2025 to January 2027) following ethics approval in January 2026.

### Patient and involvement

None.

## Ethics and dissemination

The study protocol has been approved by the Yunnan Provincial Hospital of Traditional Chinese Medicine (Approval No. 2024-KY-032-02). Written informed consent will be obtained from participants prior to enrollment. The results of this study will be published in peer-reviewed publications.

## Discussion

ICBN syndrome is a common yet easily overlooked peripheral sensory nerve injury following axillary surgery for breast cancer. Clinically, it manifests as persistent numbness and burning pain extending from the affected axilla to the medial aspect of the upper arm. Although anatomical studies confirm that the ICBN is a pure sensory nerve and its injury does not directly lead to muscle denervation, the chronic neuropathic pain (5) induced by the injury causes significant “pain-movement inhibition” (16) and kinesiophobia (17). This subsequently leads to restricted active range of motion of the shoulder joint on the affected side and secondary disuse muscle weakness (15). Its high incidence and prolonged recovery characteristics have severely affected patients’ postoperative quality of life and compliance with rehabilitation. Although “nerve preservation” has become a consensus strategy for axillary dissection, multiple studies consistently suggest that regardless of how surgical techniques are improved, a considerable proportion of patients still experience persistent sensory abnormalities for ≥12 months (5). This indicates that surgical techniques alone are difficult to completely avoid the sequelae of nerve injury. Current clinical interventions primarily consist of symptomatic analgesia and passive functional exercises, lacking standardized external treatment protocols aimed at peripheral nerve regeneration and functional reconstruction. Therefore, exploring safe, effective, and patient-acceptable external treatment schemes has become an urgent priority for improving ICBN syndrome. This study intends to conduct a prospective, randomized, sham-controlled trial to systematically evaluate the efficacy and safety of Transcutaneous TEAS for this syndrome. The goal is to establish an RCT evidence chain for TEAS intervention in post-breast cancer ICBN syndrome and provide scientific evidence-based medical evidence for clinical practice.

In this study, the CMS serves as the primary outcome measure to assess the improvement in overall shoulder joint function. The secondary outcome measures include MMT, EMG, VAS, SWMT, FACT-B, SF-36, and TCM-SD, systematically evaluating the degree of improvement in ICBN syndrome from dimensions such as subjective muscle strength, neurophysiology, pain perception, tactile threshold, quality of life, and TCM syndrome burden. Among these, MMT, EMG, VAS, and SWMT focus on sensorimotor functional recovery, and these tools have been widely validated in the field of peripheral nerve repair, demonstrating good sensitivity and reproducibility. Through dynamic observation from T0 to T3, we can quantify the time-effect relationship of TEAS on nerve regeneration, sensory recovery, and upper limb functional reconstruction, providing objective and direct evidence for clarifying its intervention effects. FACT-B, SF-36, and TCM-SD are assessed at T0 and T1 to supplementarily evaluate the impact of TEAS on patients' overall health status and TCM syndrome burden, further enriching the comprehensiveness and clinical applicability of the efficacy evaluation.

Beiji Qianjin Yaofang (Essential Prescriptions for Emergencies worth a Thousand Gold) records that “Jianzhen governs hand numbness and inability to lift”. Zhenjiu Juying (Compilation of Acupuncture and Moxibustion) states regarding Jianzhen: “…for heat and pain in the supraclavicular fossa and shoulder, and wind-bi numbness of hands and feet with inability to lift”. Zhenjiu Fengyuan (Source of Acupuncture and Moxibustion) records: “For shoulder heaviness and elbow/arm pain preventing lifting, Tianzong governs it”. Simultaneously, Jianzhen and Tianzong are distributed in the muscle spaces attached to the first and second intercostal spaces. Stimulation at these points can relax local muscles, reduce local tissue pressure, eliminate stimulation of nerve endings, promote blood circulation, and accelerate venous and lymphatic return. They are local key points for shoulder and arm pain, numbness, sensory disturbances, and functional impairment. Furthermore, since post-breast cancer ICBN syndrome involves injury to the T2 intercostal nerve, the corresponding Jiaji points above and below T2 are selected to promote the recovery of this nerve. Postoperative breast cancer patients often suffer from deficiency of Qi and blood. Zusanli is selected to regulate systemic functions; for those with physical weakness and unrecovered Qi and blood after surgery, it can invigorate the spleen and stomach, and replenish Qi and nourish blood. Sanyinjiao is the crossing point of the three Yin meridians of the foot (Taiyin, Shaoyin, and Jueyin). It can regulate and tonify the Qi and blood of the Liver, Spleen, and Kidney meridians. Simultaneously, it can correspondingly regulate the damaged three Yin meridians of the hand, playing a distal regulatory role. Therefore, the acupoints selected for this study are: Jianzhen, Tianzong, T1–T3 Jiaji, Zusanli, and Sanyinjiao.

This study strictly follows the CONSORT guidelines and the STRICTA extension for acupuncture. All source data will be uniformly recorded in the CRF and entered into the study's data management system by researchers. Double verification will be conducted before database locking to ensure accuracy. Through dynamic quality control throughout the process, the standardization of the TEAS intervention protocol and data reliability are ensured. If the results confirm that TEAS can significantly improve ICBN syndrome, it will provide scientific RCT evidence for the painless and non-invasive rehabilitation of peripheral nerve injury after breast cancer surgery, and lay a methodological foundation for subsequent research on nerve repair mechanisms. Given the advantages of TEAS, such as targeted acupoint selection, ease of operation, low cost, and high patient acceptance, once its efficacy is established, it is expected to reduce long-term use of analgesic drugs, lower medical expenses, accelerate patients' return to daily activities, and comprehensively improve the quality of postoperative rehabilitation.
